# Single-cell multiomic modelling of early metastatic events promoted by the extracellular matrix

**DOI:** 10.1038/s41416-025-03181-4

**Published:** 2025-09-15

**Authors:** Hyun Jin Lee, Jun Hyeong Lee, Junho Kang, Kyeonghui Kim, Youngwon Cho, Jihyun Park, Sang-Hyun Song, Joonha Kwon, Young-Joon Kim, Woong-Yang Park, Tae-You Kim, Jong-Eun Park, Pilnam Kim, Jung Kyoon Choi

**Affiliations:** 1 Department of Bio and Brain Engineering, KAIST, Daejeon, Republic of Korea; 2 Graduate School of Medical Science and Engineering, KAIST, Daejeon, Republic of Korea; 3 Cancer Genomics Research Laboratory, Cancer Research Institute, Seoul National University, Seoul, Republic of Korea; 4 Department of Molecular Medicine and Biopharmaceutical Sciences, Graduate School of Convergence Science and Technology, Seoul National University, Seoul, Republic of Korea; 5 Bioinformatics Branch, Division of Cancer Data Science, National Cancer Center, Goyang, Republic of Korea; 6 Department of Biochemistry, College of Life Science and Technology, Yonsei University, Seoul, Republic of Korea; 7 Geninus Inc, Seoul, Republic of Korea; 8 Samsung Genome Institute, Samsung Medical Center, Seoul, Republic of Korea; 9 Department of Internal Medicine, Seoul National University Hospital, Seoul, Republic of Korea; 10 IMBDx Inc, Seoul, Republic of Korea; 11 SCL-KAIST Institute of Translational Research, KAIST, Daejeon, Republic of Korea

**Keywords:** Cancer, Genetics

## Abstract

**Background:**

Cancer metastasis, primarily driven by epithelial-to-mesenchymal transition (EMT), is responsible for most cancer-related mortalities. Traditional pre-clinical models fail to fully capture mesenchymal characteristics due to the loss of human stroma. The extracellular matrix (ECM) plays a crucial role in EMT, yet conventional in vitro models often rely on defined ECM components, which may not adequately replicate the human physiological ECM niche.

**Methods:**

To mimic the in situ dissemination of cancer cells, we employed a patient-derived extracellular matrix (pdECM). We transitioned the culture matrix for patient-derived colorectal cancer organoids from a basement membrane extract (BME) to a patient-derived ECM (pdECM). We performed single-cell multiomic analyses, integrating transcriptomic and epigenomic data, to investigate changes in organoid phenotypes and reconstruct the EMT trajectory.

**Results:**

Organoids cultured in the pdECM exhibited increased tumor cell dissemination and motility, resembling in situ lesions without exogenous ligand treatment. Single-cell multiomic analysis revealed TNF-α signaling as an early metastatic event in the EMT trajectory. Epigenomic changes led to increased accessibility of AP-1 complex target genes, particularly MMP7, which promoted an invasive phenotype. Our multimodal computational approach distinguished early and late EMT states, demonstrating that pdECM-induced EMT occurs independently of traditional EMT master regulators. Notably, pdECM organoids exhibited a partial EMT phenotype, characterized by hybrid epithelial-mesenchymal states.

**Conclusion:**

This study presents an advanced in vitro model that closely recapitulates in situ tumorigenesis and provides novel insights into the metastatic cascade. The pdECM system enables the reconstruction of EMT dynamics, highlighting the critical role of ECM composition in metastasis and offering a physiologically relevant platform for the development of targeted therapies.

## Introduction

Cancer metastasis, the intricate process by which cancer cells disseminate from primary to secondary sites, accounts for ~90% of cancer-related mortalities [1]. Cancer cells undergo a biological phenomenon known as epithelial-to-mesenchymal transition (EMT) for metastasis. EMT is a process utilized by non-malignant cells during essential processes such as embryonic development and tissue regeneration [2]. However, cancer cells exploit it to provoke invasive behavior throughout the metastatic cascade composed of local invasion, extravasation, circulation, intravasation, and colonization [3].

Unfortunately, surgical resection is not an eligible option for most patients with incurable metastasis, impeding the biobanking of metastatic biopsy specimen for future research [4]. While traditional pre-clinical platforms using patient-derived in vivo and in vitro models reproduce most of the genetic and transcriptomic characteristics of the original samples, mesenchymal features are largely absent due to the loss of human stroma [5]. To circumvent this issue, researchers have harnessed an in vitro system that employs pro-metastatic ligands such as TGF-ß and TNF to induce EMT in cancer cells [6]. Subsequently, diverse transcription factors (TFs) including those of the ZEB, TWIST, and SNAIL family are identified as EMT master regulators [7]. However, recent studies have unveiled instances of metastasis that occur independently of the EMT master regulators in cancer organoids. For example, Flum et al. presented a compelling evidence of partial EMT (pEMT)-mediated invasion that does not involve the EMT master regulators by using TGF-ß treated colorectal organoids and highlighted the importance of microenvironmental cues in metastatic progression [8, 9].

Among the myriad factors comprising the tumor microenvironment, the extracellular matrix (ECM) has emerged as a critical contributor to the EMT of cancer cells [10]. The ECM, traditionally recognized for its role in maintaining the structural integrity of diverse tissues of the human body, also contributes to cellular signaling through both biochemical and biomechanical mechanisms [1]. Various ECM components such as type I collagen and their chemical modification such as hydroxylation govern the intricate crosstalk between cells and ECM [11]. Accordingly, numerous studies report the bidirectional interaction between cancer cells and ECM that ultimately trigger the pro-metastatic cascades and ECM remodeling, including the formation of pre-metastatic niche in diverse cancer types [12, 13]. For instance, fibrous and stiff collagen matrix in connective tissues are sufficient to drive EMT, invasion, and metastasis of cancer cells [14–16]. In fact, the connective ECMs compose over 300 core-ECM proteins and large numbers of ECM-modifying enzymes, ECM-binding growth factors, and other ECM-associated proteins [17].

The complexity of the ECM potentially impacts on cancer cells through numerous intricate mechanisms. However, previous studies have largely focused on discrete elements such as specific ECM ligand-related oncogenic pathways. Thus, the conventional in vitro system using defined ECM components requires the administration of pro-metastatic ligands that might not be fully representative of the human physiological ECM niche [8, 18]. Recent technological advancements make it possible to decellularize the tissue, extract the native ECM structure, and harness it for research and therapeutic intervention [19, 20]. This presents an opportunity to incorporate an additional layer into the in vitro EMT model.

In this study, we transition the culture matrix for patient-derived organoids from a basement membrane extract (BME) to a patient-derived ECM (pdECM). We investigate the effects of this transition on organoid phenotypes at a single-cell resolution in different ECM environments. Notably, we observe tumor cell dissemination and induction of motility, closely mirroring in situ lesions, without the need for pro-metastatic ligand treatment. To delve deeper into the underlying mechanisms, we conduct single-cell multiomic analyses and reconstruct the EMT trajectory. This analysis identifies TNF-α signaling as an early metastatic event in the EMT trajectory. Intriguingly, the cancer cells undergo genome-wide epigenome changes, leading to increased accessibility to AP-1 complex target genes. Subsequently, in the absence of basement membrane encapsulation, direct interaction with the interstitial ECM upregulates AP-1 complex target genes, including *MMP7*, which confers an invasive phenotype. Altogether, this study integrates our unique in vitro model, which recapitulates in situ tumorigenesis, with multimodal computational analysis at the single-cell level, enabling the reconstruction of the EMT trajectory. This approach provides deeper insights into the metastatic cascades of cancer.

## Materials and methods

### Tissue decellularization process and pdECM matrix preparation

Surgically collected colorectal tissues from Yonsei Severance Hospital were decellularized using a detergent-based method. The following decellularizing detergent solution was used to remove the cellular components from tissues: 1% (v/v) Triton X-100 (T8787; Sigma-Aldrich, St. Louis, MO, USA) and 0.1% (v/v) ammonium hydroxide (221228; Sigma-Aldrich) in distilled water. Tissue samples were cut into small sections (3 × 3 × 3 mm) and treated with decellularizing solution for >2 h; the solution was replaced at 30-min intervals or when it became opaque. When the tissue became colorless, the resulting pdECM samples were washed with Dulbecco’s phosphate buffered-saline (Welgene, Korea) for 2 days; the solution was replaced at 1-h intervals. Then, the tissue was washed with distilled water, 4 times for 10 min each, to remove residual Dulbecco’s phosphate buffered-saline. Decellularization was performed on an orbital shaker at room temperature, using a speed of 70 rpm. Finally, dECM samples were lyophilized for 1 day and stored at −20 °C until use. Lyophilized dECM was solubilized with 1 mg/ml pepsin (Sigma-Aldrich) in 0.01 N HCL (Sigma-Aldrich) solution for >24 h at room temperature until visible ECM particle disappeared and the final concentration of the pdECM pre-gel solution was 10 mg/ml. This study protocol was approved by the Institutional Review Board at Korea Advanced Institute of Science and Technology (KAIST), Republic of Korea (approval no.: KH2018-54), and the Severance Hospital, Yonsei University College of Medicine, Seoul, Republic of Korea (approval no.: 4-2012-0859). The study was conducted in accordance with the relevant guidelines and regulations. All participants provided written informed consent for sample collection; the study was performed in accordance with the Declaration of Helsinki.

### Proteomic analysis of the pdECM with TMT spectrometry

All decellularized colon tissues were processed using the S-Trap™ mini (ProtiFi, Huntington, NY, USA) [21] to perform protein extraction and digestion, following a slightly modified version of the manufacturer’s instructions. Peptides from each individual and pooled tissues were labeled using the TMT 11-plex (Thermo Fisher Scientific). The TMT-labeled peptides were divided into 20 fractions by using a Shimadzu HPLC system. Then, fractions were dried in a SpeedVac vacuum centrifuge and dissolved in 0.1% formic acid for LC-MS/MS analysis. A nano-flow ultra-high-performance liquid chromatography (UHPLC) system (UltiMate 3000 RSLCnano System; Thermo Fisher Scientific) coupled to the Orbitrap Eclipse™ Tribrid™ mass spectrometer (Thermo Fisher Scientific) was used for acquiring UHPLC-MS/MS data. Raw files were processed and normalized with specific parameters and the normalized protein intensities of detected protein by each sample were quantified. The detected proteins were annotated with the matrisome category and the sum of intensity values of proteins with each matrisome categories was calculated. The Pearson correlation coefficient was calculated with the intensity values of proteins included in matrisome categories between the samples.

### Culture of colon cancer organoids with the BME and pdECM

Colon cancer organoids from Seoul National University Hospital were developed with the slightly modified method as described by Sato et al. [22]. Briefly, for the initial growth, the colon cancer organoid were embedded in BME such as growth factor reduced Matrigel (354230, Corning) or Cultrex (3433-010-01 P, R&D systems) and cultured with the cancer organoid medium. The composition of medium is: Basal culture medium (advanced DMEM-F12 (12634010, Gibco) + 10 mM HEPES (15630080, Gibco) + 1 x GlutaMAX (35050061, Gibco) + 1 x Penicillin/Streptomycin (Welgene, Korea)) with 10% R-Spondin conditioned medium, 100 ng/ml hNoggin (120-10 C, Peprotech), 1 x B27 (17504044, Gibco), 1.25 mM n-Acetyl cysteine (A7250-10G, Sigma-Aldrich), 10 mM Nicotinamide (72340-100 G, Sigma-Aldrich), 50 ng/ml hEGF (238-EG-200, R&D systems), 10 nM Gastrin1 (G9020-250uG, Sigma-Aldrich), 500 nM A83-01 (SML0788-5MG, Sigma-Aldrich), 3 μM SB202190 (S7067-5MG, Sigma-Aldrich), 10 nM Prostaglandin E2 (P5640-1MG, Sigma-Aldrich), and 100 μg/ml Primocin (Ant-pm-1, InvivoGen). After the 7 days of initial growth, colon cancer organoids are subcultured with TryPLE enzyme (12605010, Gibco), re-embedded with BME or dECM pre-gel solution and seeded in 24 well cell-culture plate. To make hydrogel, in case of BME, the seeded organoid-embedded gels were incubated at 37 °C for 20 min. In case of dECM, the pre-gel solution was mixed with 10 x PBS (final concentration: 1 x PBS), and adjust to neutralized pH by adding 1 M NaOH (Sigma-aldrich) in the ice. Then, the organoids were resuspended in the neutralized pre-gel solution, seeded in the well plate and incubated at 37 °C for 30 min. To avoid the sinking of the organoid, the seeded plate was flipped after 10 min of incubation. The medium was changed with every 2–3 days. This study protocol was approved by the Institutional Review Board of Seoul National University Hospital (approval number: 1608-054-784, 1710-102-896). The study was conducted in accordance with the relevant guidelines and regulations. All participants provided written informed consent for sample collection; the study was performed in accordance with the Declaration of Helsinki.

### Imaging the morphology of the BME-O and pdECM-O

The structure and morphology of cultured organoids were observed with the optical microscope and confocal microscope. After the subculture, the growth of organoid was tracked by phase imaging with optical microscope (Leica). Relative growth of organoids was measured with the relative covered area compared with the covered area on 1 day after subculture. After 7 days of subculture, cultured organoids were fixed with 2% paraformaldehyde solution (PFA) + 0.1% glutaraldehyde (BME) or 4% PFA (pdECM) and permeabilized with 0.5% Triton X-100. DAPI (1:2000) and phalloidin (1:500) were treated to stain the nucleus and actin filament. Immunofluorescence image of cultured organoids was obtained with the confocal microscope (Nikon Eclipse Ti2). All images were processed by imageJ/FIJI program.

### Bulk transcriptome analysis

The cultured organoids were harvested and maintained in TRIzol reagent for bulk tissue RNA-seq. The indexed cDNA sequencing libraries were prepared from RNA samples using the TruSeq Stranded mRNA LT Sample Prep Kit. Quality control analyses of RNA integrity number and rRNA ratio were performed using the 2200 TapeStation. The indexed libraries were prepared as equimolar pools and sequenced on the NovaSeq 6000 to generate a minimum of 60 million paired-end reads per sample library. The raw Illumina sequence data were demultiplexed and converted to fastq files. Then, the adaptor and low-quality sequences were trimmed. The mRNA sequencing reads were mapped to Homo sapiens genome assembly GRCh37 from the Genome Reference Consortium by HISAT2 (v.2.1.0). Mapped reads were assembled with known genes and quantified in terms of read counts and sample normalized values, such as fragments per kilobase of transcript per million mapped reads and transcripts per million mapped reads (TPM), using StringTie (v.2.1.3b). Differentially expressed (DE) genes were obtained by calculating fold change between two culture condition and filtering with minimum normalized TPM values. The genes were excluded for DE genes when the TPM values of genes in all condition were lower than 50. In the both organoids (organoid A and B), if the fold change of TPM values of the gene between two condition (BME, pdECM) is bigger than 1.4, the gene was considered as DE genes. The expression patterns of specific gene sets in each cultured organoid were evaluated using single sample gene set enrichment analysis (ssGSEA). The ssGSEA scores for gene sets associated with WNT targets [23], cancer stem cell [24], epithelial, mesenchymal feature [25], EMT activation [26], wound response (GO_BP), immune infiltration (GO_BP), JAK-STAT (KEGG), VEGF, VEGFR (R-HSA-194138), invasion signature [27], degradation of ECM (R-HSA-1474228) were calculated using the ssGSEAprojection package in the GenePattern web-based tool.

### Single-cell multiomic sequencing of cultured organoids

Cultured organoids in BME or dECM were washed with DPBS and harvested with treatment of trypsin/EDTA 0.25% (Welgene, Korea) for 30 min at 37 °C. After the treatment, the cell suspension was filtered through 40μm Flowmi Cell Strainer. For the multiome sequencing, the nuclei of filtered cells were isolated with the demonstrated protocol provided by 10× genomics (Documentation #: CG000365). The isolated nuclei were passed quality-control and proceeded to the library construction. The Chromium Next GEM Single-Cell Multiome Library and Gel Bead Kit (10X Genomics) was used according to the manufacturer’s protocols (Documentation #: CG000338). Constructed libraries were sequenced on an Illumina Novaseq 6000 sequencer.

### Pre-processing of single-cell multiomic sequencing data

The raw fastq files of 10X Chromium single-cell multiomic sequencing were processed with cellranger-arc (v.1.0.1) using GRCh38 as reference genome with default parameters. Quality control was performed separately for single-cell RNA sequencing (scRNA-seq) and single-cell ATAC sequencing (scATAC-seq) using Seurat (v.4.2) [28] and SnapATAC (v.1.0.0) [29]. First, to pre-process scRNA-seq data, genes expressed in less than 10 cells were filtered out. Then, we selected cells that passed these following criteria: (i) 1000 < number of genes <10,000, (ii) 1000 < number of UMI < 100,000, (iii) percentage of mitochondria <50%, and (iv) log_10_(number of genes per UMI) > 0.8. For scATAC-seq data, we filtered for cells that met these following criteria: (i) 0.25 < promoter ratio <0.8, and (ii) 1000 < UMI count <100,000. Then, we filtered out fragments that overlapped with blacklist of hg38 or originated from mitochondrial chromosome. Only the cells that passed both scRNA-seq and scATAC-seq filtering steps were kept for the analysis.

### Analysis of single-cell multiomic sequencing data

We employed Seurat [28] and SnapATAC [29] to process and analyze the transcriptomes and epigenomes of the multiome data. For scRNA-seq analysis, we performed log normalization with scale factor of 10,000, and linear transformation to set the mean expression as 0 and the variance as 1. Then, we identified variable features to determine the principal components (PC). Subsequently, the PCs were used to project the cells into Uniform Manifold Approximation and Projection (UMAP) or t-Distributed Stochastic Neighbor Embedding (tSNE) space. DE analysis was conducted using *FindMarkers* to identify genes with log_2_(fold change) >0.25, and expressed in more than 25% of the cells. The enrichment scores of hallmark states (e.g., MSigDB Hallmark TNF-α signaling) of cells were calculated using *AddModuleScore*. We used WNT in Epithelial to Mesenchymal Transition in Cancer from Elsevier database to calculate EMT score. For replicate 1 and 2, we used WNT in Epithelial to Mesenchymal Transition in Cancer from Elsevier database and Hallmark EMT from MSigDB Hallmark, respectively. Clusters of cells were labeled as late if EMT score is higher and early if EMT score is lower. To conduct scATAC-seq analysis, we first computed a diffusion map, identified significant components, and visualized the cells in UMAP or tSNE space. Peak calling was performed using *runMACS*. The peaks from pdECM-O and BME-O cells were merged to yield a combined peak profile. Top 2000 most differentially accessible regions were identified with *findDAR* using merged peaks. The motif enrichment and variability of TFs was evaluated using *runHomer* and *runChromVAR* with default parameters, respectively. To integrate two data modalities, we employed Weighted-Nearest Neighbor (WNN) algorithm that is implemented in the Seurat package. Briefly, this algorithm determines the relative contribution of each data modality—transcriptome and epigenome in this study—on a per-cell basis. Each modality is then preprocessed and subjected to dimensionality reduction. Finally, the WNN workflow integrates these modalities by assigning weights to each data type, constructing a WNN graph, and projecting the cells into a unified multimodal space [28]. The WNN graph was constructed with *FindMultiModalNeighbors* and the cells were embedded in the multiome space with UMAP. To account for patient-specific characteristics of epithelial cells, the samples were analyzed separately.

### Reference mapping analysis using clinical cancer tissue and multiome data

To examine whether in vitro pdECM system genuinely reflects clinical cancer tissues, we conducted reference mapping analysis using clinical cancer tissue and our multiome data. To process clinical cancer tissue data, we first curated unsorted malignant and non-malignant colon tissue scRNA-seq generated with 10X chemistry in public data repositories, including (i) PubMed, (ii) Google Scholar, (iii) Gene Expression Omnibus, (iv) Single Cell Portal, (v) COVID-19 Cell Atlas, and (vi) Curated Cell Atlas. Then, the cells that met these following criteria were retained: (i) 2000 < UMI count, (ii) 500 < number of genes detected <7000, and (iii) not called as doublet in Scrublet [30]. This resulted in 7 datasets, 62 cases, and 253,475 cells. The datasets were batch-corrected with BBKNN and harmony [31, 32] and the cells were annotated in UMAP space based on well-known markers. Only the genes of GRCh38 that were found across the datasets were retained. Then, we mapped epithelial cells of primary colon cancer tissues with metastasis from clinical cancer tissue data to our multiome data. To do so, we first normalized and embedded multiome-sequenced cells with *SCTransform* and computed supervised PCA using *RunSPCA*. Then, we identified transfer anchors using *FindTransferAnchors* and subsequently transferred labels of reference (pdECM/BME-O cells) to query (epithelial cells of primary colon cancer tissues with metastasis from clinical cancer tissue data) using *MapQuery*. We then calculated the proportion of epithelial cells that mapped to the pdECM/BME cells to evaluate whether the pdECM cells well reflect clinical tissues.

### Identifying promoters and associated distal regulatory elements

The promoters of the genes are set as ±0.5kbp transcription start site defined in gencode v37 [33]. We then determined regulatory elements (REs) that are dispersed 1Mbp around the promoters with a regression model (*predictGenePeakPair*). These promoters and REs were used to quantify the accessibility of the promoters and REs of the genes. To visualize dynamics of the genes at the multiome level, we employed LOESS smoothing along the EMT trajectory.

### Functional enrichment analysis

To evaluate the biological annotation of the genes (e.g., differentially expressed genes between the pdECM-O and BME-O cells), we employed EnrichR [34]. Only the terms with adjusted *p*value < 0.05 were considered significant.

### EMT trajectory analysis

To conduct trajectory analysis in the multiome space, we provided multiome-UMAP coordinate of the cells into Monocle3 [35]. We selected cluster of cells with lower EMT score as starting point of the trajectory and identified genes that are differentially expressed along the trajectory. To only retain genes that are consistently and differentially expressed along the trajectory, we first selected genes with adjusted *p*value < 0.05 in Monocle3. Then, we calculated Pearson correlation between pseudotime and expression of the genes and retained genes with correlation coefficient >0.15.

### MMP7 concentration and inhibition analysi*s*

MMP7 concentration of organoid cultured media was analyzed with Human Total MMP-7 Quantikine ELISA kit (DMP700, R&D systems) according to the manufacturer’s protocol. The conditioned media of organoids was harvested after 7 days culture on each platform (BME, pdECM). To inhibit the effect of MMP7, 10 μM of batimastat (BB-94, Selleckchem) was supplemented with the complete organoid media.

## Results

### pdECM replicates changes in cell-matrix interaction in organoid culture

**Fig. 1:**
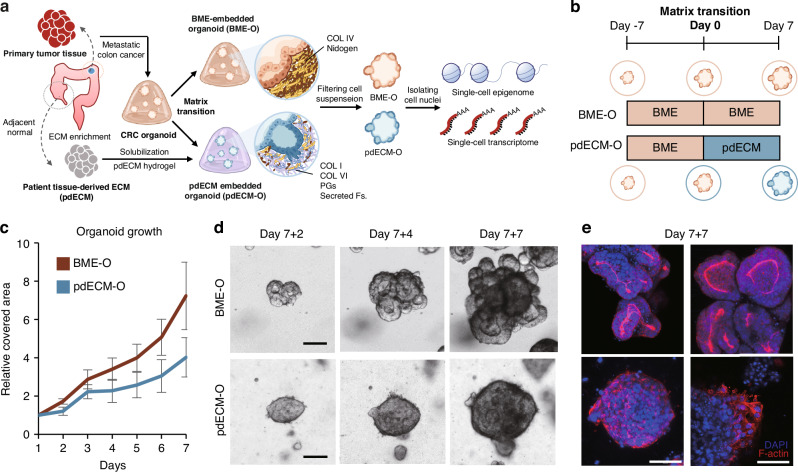
Human colon cancer organoid culture in human-tissue-derived decellularized ECM. **a** Overall scheme of our research. Human colon cancer organoids were cultured both in the BME and pdECM. Cultured organoids were analyzed by single-cell multiomic sequencing. **b** Flow chart of our organoid culture involving matrix transition. **c** Organoid growth in the BME and pdECM by measuring relative covered area. Standard deviation was expressed in the graph. **d** Representative images of organoids cultured in the BME and pdECM. Scale bar. 100 μm. **e** Representative images of nucleus and actin filament of BME-O and pdECM-O. Scale bar. 100 μm. **a**–**e** BME basement membrane extract, BME-O BME-cultured organoid, pdECM patient-derived extracellular matrix, pdECM-O pdECM-cultured organoid.

Cancer organoids, isolated from primary tumor tissues of patients with metastatic colon cancer, were used to model basement membrane disruption during the initial stage of metastasis. To achieve this, we transitioned the culture matrix from a BME (Matrigel®) to a pdECM. Organoids cultured in these different matrices were then subjected to single-cell multiomic analysis to investigate their molecular features in detail, encompassing both the epigenome and transcriptome (Fig. 1a).

We constructed a pdECM using a modified version of our previously method [36]. Tandem mass tag (TMT) technology was employed for the quantitative analysis of pdECM protein components. Our proteomic analysis of pdECMs from seven normal tissues revealed a high degree of consistency among the samples (Fig. S1A). Notably, the pdECM proteome composition was distinct from the well-established protein components of the BME [37]. Unlike the BME, which is primarily composed of basement membrane components, the pdECM exhibited a significant presence of interstitial matrix components, such as collagen types I and VI (Fig. S1B). To mitigate batch effects and enhance experimental reproducibility, we prepared a sufficient quantity of the pdECM mixture for organoid culture by combining tissues from 16 individual patients.

Organoids were initially cultured in the BME for 7 days to facilitate initial growth. Subsequently, a subset of the organoids was transferred to the pdECM and cultured for additional 7 days (Fig. 1b). The growth of the BME-cultured organoid (BME-O) was faster than that of the pdECM-cultured organoid (pdECM-O; Fig. 1c). The BME-O displayed a typical luminal structure in contrast to the pdECM-O, which showed an irregular form with cells protruding from the organoid periphery (Fig. 1d). Actin filament staining revealed the differences of actin polarity and protrusions in border (Fig. 1e). Intriguingly, in the pdECM-O, the actin polarity of the organoid underwent an inversion from an apical-in pattern to an apical-out pattern. This observation indicates that the interaction between the organoid and the matrix markedly influences cell phenotypes, highlighting that interaction with the interstitial ECM prompts a conversion in epithelial cell polarity.

### pdECM organoids undergo EMT with an invasive phenotype

**Fig. 2:**
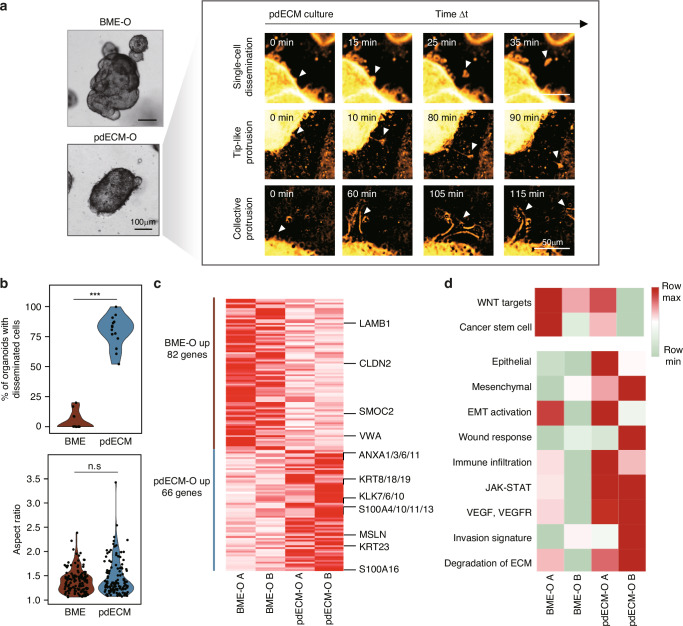
Invasive phenotype and gene expression profile observed in the pdECM culture. **a** Representative images of invasive phenotype observed in the pdECM culture. Single-cell dissemination, tip-like protrusion, and collective protrusion were observed in the pdECM-O. The images were pseudo-colored from black/white phase image. **b** Violin plot of the percentage of organoids with disseminated cells and the aspect ratio of organoids measured in each culture condition. *** : *p* < 0.001. **c** Heatmap of genes that were differentially expressed between the BME-O and pdECM-O. Major EMT-related genes were annotated. Two replicate experiments (**a** and **b**) were included in the analysis. **d** Heatmap of gene set enrichment scores for differentially expressed genes between the BME-O and pdECM-O. Two replicate experiments (**a** and **b**) were included in the analysis.

The pdECM-O frequently exhibited an invasive phenotype, a characteristic not observed in the BME-O. Time-lapse recordings of BME-O and pdECM-O growth revealed three distinct invasion-related movements: the dissemination of individual cells, the formation of tip-like protrusions, and the emergence of collective protrusions (Fig. 2a and **Supplementary Movies**). In the pdECM culture, we observed not only highly motile individual cells but also collective migrations led by a leader cell. Upon measuring the morphological changes induced by the matrix, we found a greater number of disseminated cells in the pdECM-O (Fig. 2b). These findings suggest that direct contact with the pdECM promotes the initiation of EMT in cancer organoids.

In addition to morphological differences, we compared the transcriptomic profiles of the BME-O and pdECM-O. Differential expression analysis revealed the upregulation of genes associated with tumor invasiveness in the pdECM-O. This includes the annexin family (ANXA) [38], the kallikrein family (KLK) [39, 40], the S100A family [41], mesothelin (*MSLN*) [42], and keratin 23 (*KRT23*; Fig. 2c) [43]. Concurrently, there was a notable decrease in claudin 2 (*CLDN2*; Fig. 2C) [44]. Single-sample gene set enrichment analysis indicated the downregulation of WNT signaling and stem cell-associated genes in the pdECM-O (Fig. 2d). Conversely, genes related to mesenchymal traits, EMT activation, VEGF signaling, invasion signatures, and ECM degradation were overexpressed in the pdECM-O (Fig. 2d). This data suggests that transitioning to the pdECM prompts a transformation in the molecular characteristics of the organoids, promoting an invasive phenotype not observed under conventional BME-based culture conditions.

### Multimodal single-cell clustering of pdECM and BME organoids

We next sought to explore the impact of embedding in the pdECM on the phenotypic heterogeneity of cancer at single-cell resolution. To this end, we performed single-cell multiomic sequencing on the pdECM-O and BME-O to elucidate heterogeneity at both the transcriptional and epigenetic levels. Weighted Nearest Neighbor (WNN) algorithm [28] was employed to project the BME-O and pdECM-O cells onto a multimodal dimension.

**Fig. 3:**
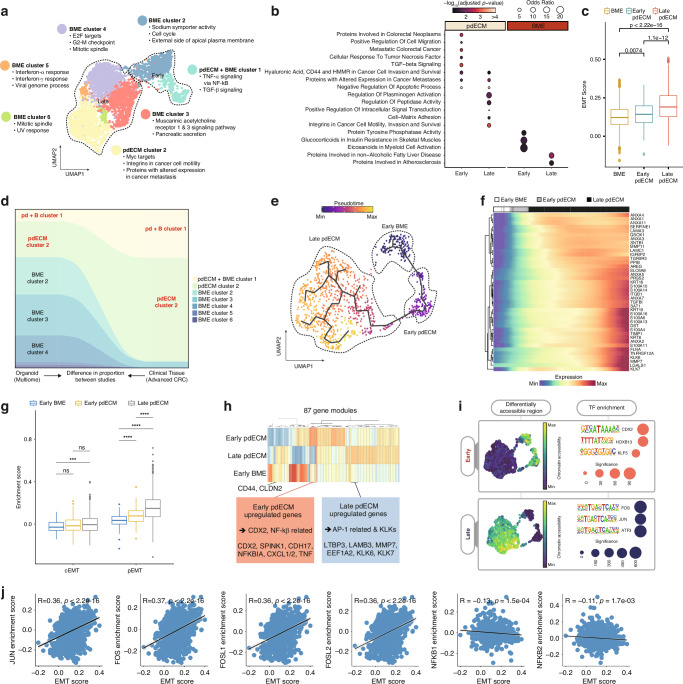
Multimodal dissection of the pdECM-O at single-cell resolution. **a** Identification of BME and pdECM clusters in the multiome space. The cells were projected to the multiome UMAP space using the WNN algorithm. Then, the clusters were biologically annotated using Enrichr. UMAP uniform manifold approximation projection, WNN weighted nearest neighbor. **b** Biological annotation of genes upregulated in the early/late pdECM/BME-O cells. Marker genes of each cluster were annotated with Enrichr using GO Molecular Functions, GO Biological Process, and Elsevier Pathway Collection libraries. The size and color of the circles represent the odds ratio and *p*value of each term, respectively. **c** EMT scores of the BME-O, early pdECM-O, and late pdECM-O cells. Statistical differences were calculated with the *t* test. **d** Alluvial plot depicting the proportion of the pdECM/BME clusters and that of epithelial cells from clinical colon cancer tissues that map through reference mapping analysis. **e** Pseudo-ordered cells along the EMT trajectory. The cells were ordered and colored according to their EMT scores. **f** Expression of EMT-related genes including annexin, S100A, keratin, and metallopeptidase family genes along the EMT trajectory. Each row and column represent a gene and pseudo-ordered cell, respectively. For visualization, log-normalized expression level of the cells was spline-smoothened and zero-centered with unit variance. **g** cEMT and pEMT scores of the early BME, early pdECM and late pdECM cells. Difference in the EMT score was calculated with the *t* test. * denotes pairs with *p*value < 0.05. **h** Hierarchically clustered heatmap of gene modules obtained from Monocle analysis. **i** (Left) DARs between the early BME-O/pdECM-O cells and the late pdECM-O cells. The colors represent the DAR accessibility of the cells. (Right) TF-binding motifs in the DARs were identified using HOMER. The size represents the adjusted *p*value of motif enrichment. **j** Spearman correlation between the TF enrichment score and EMT scores. Each dot represents a single cell. Black line represents the line that best fits the data distribution and the gray denotes the confidence interval. The *p*value is adjusted with the Benjamini-Hochberg method.

This integrative approach that combines transcriptomic and epigenomic data revealed multiple distinct clusters with unique biological functions, separating the pdECM-O cells from their BME-O counterparts in the multiome space (Fig. 3a). The only cluster containing both pdECM-O and BME-O cells was the pdECM+BME cluster 1. Our functional analysis revealed that pdECM+BME cluster 1 was characterized by TNF-α and TGF-β signaling, while pdECM cluster 2 was associated with metastasis-relevant gene expression programs (Fig. 3a). In contrast, clusters derived from the BME-O displayed biological functions unrelated to invasion or EMT.

We hypothesized that pdECM+BME cluster 1 reflects an early state prior to the divergence between the pdECM and BME cultures. Therefore, pdECM+BME cluster 1 and its neighbor, BME cluster 2, were collectively labeled as early, while the other clusters were labeled as late. In fact, the early and late clusters exhibited markedly different gene expression patterns (Fig. 3b). In particular, the pdECM-O cells at later stages (i.e., pdECM cluster 2) were represented by functional terms such as cell-matrix adhesion, cancer cell motility, and cell invasion and survival. Also, the late pdECM-O cells showed significantly higher EMT scores than both the early pdECM-O cells and the BME-O cells (Fig. 3c).

We then examined whether the early and late stages could be distinguished only when analyzing the pdECM-O cells after excluding the BME-O cells. When projected onto the transcriptome, epigenome space, and multiome space, the early pdECM-O cells and the late pdECM-O cells were distinctly separated (Fig. S2A). For validation, we repeated the experiments and analyses using two biological replicates from different organoid samples (replicates 1 and 2). As a result, multiomic clustering enabled clear and robust discrimination between the early and late pdECM-O cells, as annotated by their EMT scores (Fig. S2B). However, epigenomic profiles alone were insufficient to fully resolve the two states in replicate 2, illustrating the importance of a multiomics approach for capturing unique cellular states and elucidating their functional characteristics. Additionally, the upregulation of EMT-related genes in the late pdECM-O cells was recapitulated in the replicates (Fig. S2C).

Additionally, we sought to determine whether the invasive phenotype of the pdECM-O accurately reflects the EMT process observed in in situ lesions. To address this, we collected single-cell transcriptome data from 62 clinical tissues of colorectal cancer. Through our reference mapping analysis, we found that the majority of epithelial cells from these clinical tissues closely corresponded to the cells of the pdECM+BME cluster 1 and pdECM cluster 2 (Fig. 3d). This comparison suggests that our pdECM-based organoid culture more accurately recapitulates in situ tumor lesions than conventional BME-based culture settings.

### Multimodal analysis according to the EMT trajectory

The pdECM-O cells, and the BME-O cells assigned to cluster 1 (Fig. 3a), were subjected to downstream analyses, as they closely resembled the characteristics of clinical samples. Pseudo-time ordering divided them into three distinct clusters, annotated as the early BME, early pdECM, and late pdECM (Fig. 3e). We analyzed EMT-related gene expression along the pseudo-time trajectory and observed a distinct upregulation of annexin, keratin, and kallikrein family genes (Fig. 3f and Fig. S3 left). S100A family genes, which are involved in the dissemination of metastatic colon cancer cells [45], were also differentially expressed along the trajectory (Fig. 3f and Fig. S3 right).

We also used cancer hallmark genes to evaluate different modes of EMT [46]. In particular, we included pEMT and complete EMT (cEMT) markers. pEMT indicates an intermediate hybrid state in which both epithelial and mesenchymal markers are coexpressed [47], whereas cEMT signifies a full acquisition of the mesenchymal phenotype. In our analysis, the pdECM-O cells aligned along the EMT trajectory showed an increase in the pEMT state with no considerable sign of cEMT (Fig. 3g). This is in line with a recent report of pEMT-triggered metastatic dissemination and colonization of cancer organoid cells and relatively insignificant role of cEMT [48]. These findings indicate that the migratory phenotype of the pdECM-O cells can be attributed to pEMT-mediated dissemination.

For more detailed functional analyses, we utilized Monocle [35] to identify differentially expressed gene modules among the cell clusters (Fig. 3h). In total, 87 gene modules with similar gene expression patterns were identified. Notably, genes associated with CDX2 and NF-kB pathways, including *CDX2*, *CDH17*, *NFKBIA*, and *CXCL1*/*2*, were specifically expressed in the early pdECM state. Conversely, genes related to the AP-1 transcriptional complex, such as *LTBP3*, *LAMB3*, *MMP7*, and *KLKs*, were overexpressed in the late pdECM-O. This analysis highlights the activation of distinct gene expression programs at different stages of the pdECM culture, thereby shedding light on the evolving transcriptional landscape during the EMT process.

We validated these transcriptional findings at the chromatin level. Differentially accessible regions (DARs) identified from our single-cell multiomic data showed distinct TF binding patterns across the genome (Fig. 3i). The DARs of the early pdECM cells were overrepresented by binding sites for *CDX2*, *HOXB13*, and *KLF5* (Fig. 3i). CDX2 and NF-kB pathway genes were included in the early transcriptional programs (Fig. 3h). In contrast, the DARs of the late cells were enriched for binding sites for AP-1 family TFs, such as *FOS*, *JUN*, and *ATF3* (Fig. 3i), in line with the overexpression of AP-1 target genes in the late pdECM state (Fig. 3h). Moreover, the binding motifs of the AP-1 complex subunits were significantly associated with the EMT score (Fig. 3j and Fig. S4A, B). In contrast, well-known EMT master regulators, such as *ZEB1*, *SNAI2*, and *MYC*, showed no evident accessibility and association with the EMT state (Fig. S4C, D). This is in line with a recent study that reports EMT processes occurring independently of these well-known EMT master regulators [8].

### Activation of TNF-α pathway genes and MMP7 according to EMT phases

**Fig. 4:**
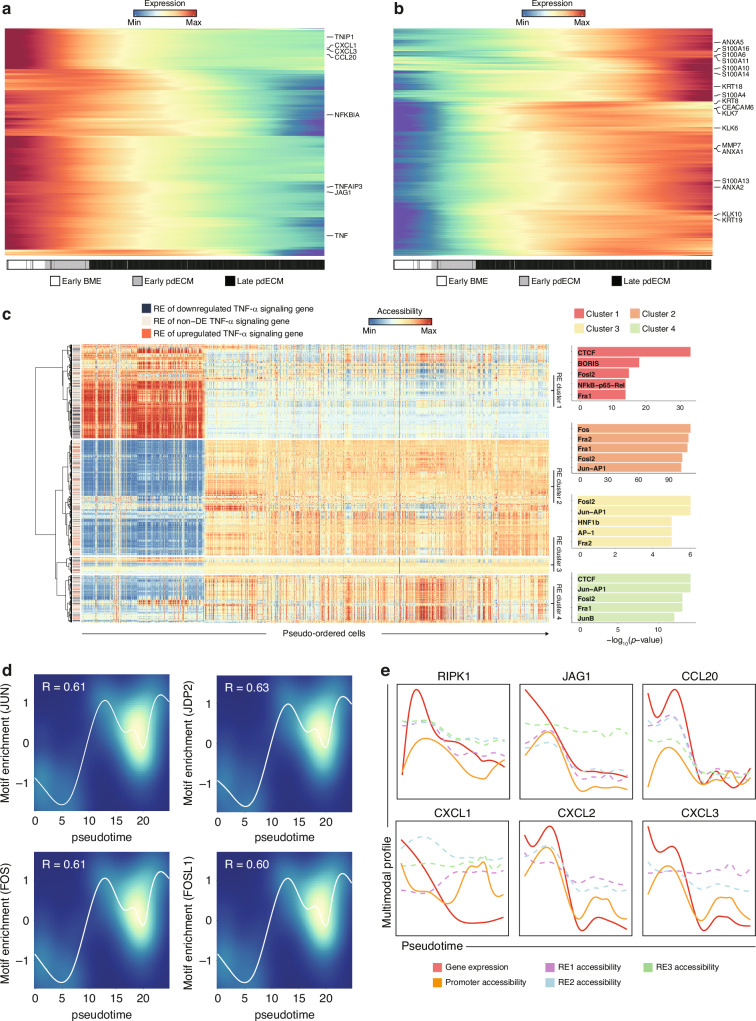
Transcriptional regulation of TNF-α pathway activity along the EMT trajectory. **a** Expression of gradually decreased genes along the EMT trajectory. Rows and columns represent genes and pseudo-ordered cells, respectively. Denoted genes represent TNF-α signaling pathway genes. For visualization, log-normalized expression level of the cells was spline-smoothened and zero-centered with unit variance. **b** Expression of gradually increased genes along the EMT trajectory. Rows and columns represent genes and pseudo-ordered cells, respectively. Denoted ones represent genes in diverse family such as annexin, S100A, keratin, kallikrein and metallopeptidase. For visualization, log-normalized expression level of the cells was spline-smoothened and zero-centered with unit variance. **c** (left) Dynamics of the REs of TNF-α signaling pathway genes. Rows and columns represent the REs and pseudo-ordered cells, respectively. For visualization, RE accessibility was zero-centered with unit variance. The REs were grouped into four clusters with hierarchical clustering using Ward.D2 distance. (right) Bar plot represents TF motif enrichment of RE clusters. The *p*value was obtained from HOMER and adjusted with the Benjamini-Hochberg method. **d** TF motif enrichment along the EMT trajectory. LOESS was used to regress the line, and the Spearman correlation was used to calculate TF motif enrichment according to the pseudo-time trajectory. **e** Dysregulation of TNF-α signaling genes at the transcriptional and regulatory level. Multimodal profiles (i.e., gene expression and chromatin accessibility) of the cells were zero centered with unit variance. The lines were smoothened with generalized additive models.

We performed differential expression analysis along the pseudo-time trajectory reconstructed from our multiomic data. Declining expression was primarily characterized by genes involved in TNF-α signaling (e.g., *RIPK1*, *CCL20*, *TNF*, and *CXCL3;* Fig. 4a), whereas increasing expression was enriched for genes involved in the EMT process (e.g., *MMP7*, *KLK7*, *TGFBI* and *ITGB1*; Fig. 4b). While inactive in the early BME-O cells, the TNF-α and TGF-β pathways were activated in the early pdECM-O cells, despite the absence of pro-metastatic ligand treatment (Fig. S5A). These two pathways showed a strong co-expression pattern (Fig. S5B). This implies that the core matrisome of the pdECM harbors multiple essential ligands for initiating signal transduction in cancer cells, further highlighting the value of the pdECM in in vitro modeling of cancer metastasis.

To further explore the regulation of TNF-α signaling, we identified regulatory elements (REs) that are co-accessible with the promoter of TNF-α signaling genes and subsequently analyzed their TF binding patterns. This analysis delineated four distinct RE clusters for these genes (Fig. 4c). Of note, RE cluster 1, enriched for TFs such as CTCF and NF-kB, was primarily accessible in the early phase of the EMT trajectory. In contrast, the other RE clusters, becoming accessible in the later phase of the trajectory, were characterized by binding motifs of the AP-1 complex subunits. In fact, across the whole genome, motif enrichment for AP-1 complex subunits was evident along the EMT trajectory (Fig. 4d). We also observed both transcriptional and epigenetic changes in key genes of the TNF-α pathway genes implicated in initiating EMT (Fig. 4e) [49–51]. Previous studies indicate that while NF-kB and AP-1 are central signaling axes in the TNF-α pathway, they are typically incapable of dynamically remodeling chromatin structure [52]. Therefore, our findings collectively suggest that the pdECM influences the TNF-α pathway not only by sequentially activating NF-kB and the AP-1 complex but also by triggering widespread epigenetic alterations. This leads to differential accessibility of NF-kB and AP-1 target genes in the TNF-α pathway.

**Fig. 5:**
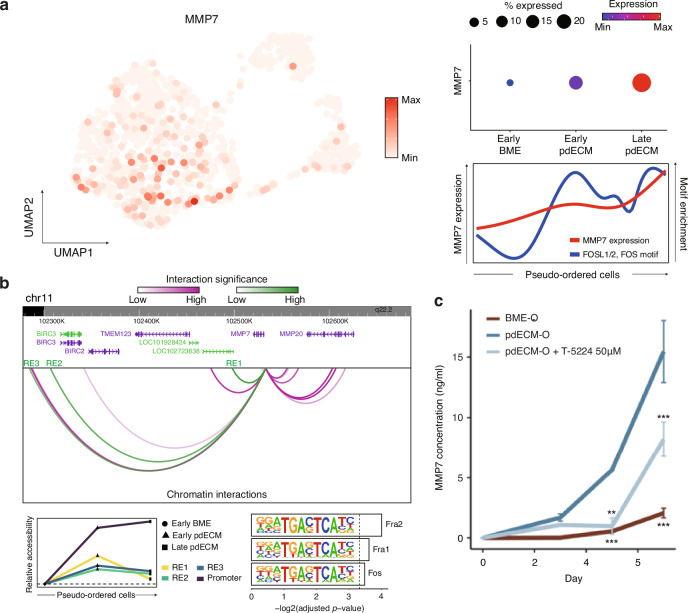
Single-cell multiomic analysis of MMP7. **a** (left) Expression of *MMP7* in the multiome UMAP space. The color represents the expression level of *MMP7*. (top right) Dotplot depicting the expression level of discrete EMT stages. The color and size represent expression level and proportion of cells expressing, respectively. (bottom right) Smoothened line depicting the expression level of *MMP7* and TF motif enrichment along the EMT trajectory. The lines are smoothened with a generative additive model. **b** (top) Chromatin interaction profile of *MMP7*. Arcs represent the REs associated with the promoter of *MMP7*. Green represents the REs that are more accessible in the late pdECM cells, and magenta denotes the remaining REs. The REs that overlap with the promoters of other genes or fail to meet statistical significance (adjusted *p*value from regression model <0.05) were filtered out. (bottom left) Relative accessibility of the REs that become more accessible in the pdECM-O cells. Relative accessibility was defined as the average accessibility of the REs in the early BME-O, early pdECM-O, and late pdECM-O cells divided by the average accessibility of the REs in the early BME-O cells. The dotted line represents where relative accessibility is 1. (bottom right) TF motif enrichment of the REs that are more accessible along the EMT trajectory. The dotted line represents where adjusted *p*value is 0.1. **c** MMP7 protein concentration of conditioned media of each culture platform. The concentration of MMP7 was analyzed from the conditioned media harvested in 3, 5, 7 days of culture from each culture platform. T-5224 (AP-1 inhibitor, 50 μM) was used for inhibiting the effect of the AP-1 complex (*** : *p*value < 0.001).

Our integrative single-cell analysis revealed a considerable upregulation of *MMP7*, a well-established facilitator of ECM breakdown crucial for cancer invasion and metastasis [53], along the EMT trajectory (Fig. 5a). Moreover, *MMP7* expression showed a marked correlation with motifs of TFs such as FOS and FOSL1/2 (Fig. 5a and Fig. S6). Consistently, the REs of the *MMP7* promoter, exhibiting increased accessibility along the EMT trajectory, were enriched for motifs of the AP-1 complex subunits (Fig. 5b). These findings indicate that the pdECM ultimately upregulates AP-1 target genes, resulting in the transcriptional activation of *MMP7* and subsequent cancer cell invasion.

The upregulation of MMP7 in the pdECM-O was validated at the protein level through the quantification of soluble MMP7 protein concentration in the cultured media using the enzyme-linked immunosorbent assay method. As a result, MMP7 protein was found to be specifically overexpressed in the pdECM-O (Fig. 5c). Interestingly, when we treated AP-1 inhibitor (T-5224, 50 μM) to the pdECM-O, the expression of MMP7 was significantly decreased. These results demonstrate that the downstream protein MMP7 could be regulated by AP-1 inhibition. Additionally, to further establish the role of MMP7 in the invasive properties of the pdECM-O, we utilized an MMP inhibitor (MMPi). Upon MMPi treatment, cells embedded in the pdECM exhibited no signs of dissemination, in stark contrast to the clear cell dissemination observed in the untreated control group (Fig. S7). Overall, these results provide insights into the role of the pdECM in regulating the transcriptome and epigenome during EMT. They underscore the importance of an integrative approach using single-cell multiomic sequencing in such studies.

## Discussion

Over the last decade, our understanding of the EMT process has significantly expanded. Unlike other physiological processes hijacked by cancer cells, EMT can be considered a pathological phenomenon that drives cancer progression, specifically influencing invasion and metastasis [54]. Our multimodal analysis suggests that the native ECM structure of a relevant organ can spontaneously induce EMT in patient-derived organoids. We observed that organoids in contact with the native interstitial ECM undergo chromatin structural remodeling, leading to the activation of diverse signaling pathways. This underlines the critical role of the ECM environment in modulating cellular behavior and underscores the complexity of cancer progression mechanisms.

Recent studies have actively explored pEMT states in cancer cells using multi-omic analyses. These investigations have identified key modulators of pEMT under various environmental cues, such as exogenous stimulation with TGF-β or stress conditions like nutrient deprivation [55, 56]. While such approaches have provided valuable mechanistic insights with multi-omics technologies, many of these studies rely on artificial induction of EMT in 2D culture systems, which lack the biophysical and biochemical interactions present in the native tumor microenvironment [57].

In contrast, our study offers a distinct perspective by examining pEMT induction within a 3D organoid culture system embedded in pdECM, without the need for exogenous EMT-promoting signals. We demonstrate that changes in the ECM composition alone are sufficient to induce an invasive phenotype that is rarely observed in conventional BME-based cultures. Through integrated multi-omic profiling, we show that a subset of cells within the pdECM environment undergoes a spontaneous transition toward a pEMT state. Notably, we identify exclusive upregulation of ECM-degrading enzymes such as MMP7, which is absent in BME cultures, suggesting that specific matrix cues promote a functionally distinct transcriptional program. This system thus provides a valuable platform to track EMT-related gene expression dynamics along pseudotime and to investigate functional heterogeneity among disseminated versus non-disseminated cells. Moreover, spatial transcriptomics approaches, such as Xenium, could be applied to further resolve functional gene programs in phenotypically distinct cancer cell subpopulations.

Various studies have linked cellular stress factors such as hypoxia and nutrient deprivation to pEMT [58]. Thus, delving into cancer hallmark states that thrust malignant cells into this transitional phase presents another avenue for therapeutic intervention. Recent years have seen systematic characterization of hallmark features across various cell types [46, 59]. Moreover, investigating the cellular origins of ligands such as TNF and TGF-β, which are pivotal in triggering the metastatic cascade, and their interactions with metastatic colon cancer cells, is crucial. The identification of TNF-α signaling as an early EMT event further underscores the complexity of ECM-mediated signaling in tumor progression. Our single-cell multiomic analyses reveal that the pdECM activates transcriptional programs involving the AP-1 complex, with downstream targets like MMP7 playing key roles in promoting cancer cell invasion. This activation is accompanied by genome-wide epigenetic remodeling, highlighting the dynamic interplay between the tumor microenvironment and the cancer epigenome. The enrichment of AP-1 motifs in chromatin accessible regions, coupled with functional inhibition experiments, validates the role of this transcription factor complex in regulating MMP7 and driving ECM degradation. These findings extend our understanding of how the ECM not only provides structural support but also acts as a critical regulator of signaling pathways that drive cancer metastasis.

Our study also addresses the broader implications of using the pdECM in cancer research. The ability to generate the pdECM from patient-derived tissues introduces a physiologically relevant model to study cancer metastasis. Unlike conventional ECM substitutes like BME, which lack the complexity and composition of human interstitial ECM, the pdECM captures the heterogeneity and biochemical cues present in the native tumor microenvironment. This makes it possible to investigate diverse stages of the metastatic cascade. In this study, we focused specifically on the impact of the pdECM derived from colon tissues on the phenotype of colon cancer organoids. Although the study did not perform large-scale systemic screening of all pathways related EMT, based on our findings, we suggest that pharmacological inhibition of key drivers such as the AP-1 complex could mitigate cancer cell invasion, providing a rationale for exploring these pathways as therapeutic targets. Likely, leveraging the pdECM to investigate drug resistance mechanisms in metastatic cancer cells could lead to the identification of novel vulnerabilities that arise during EMT. The potential of our experimental framework extends beyond colon cancer. Particularly, it would be intriguing to examine how the pdECM derived from common metastatic sites such as the liver and lungs [60] impact colon cancer organoids.

Finally, the scalability of pdECM-based systems for high-throughput applications presents exciting opportunities for broader cancer research. By incorporating omics technologies, such as proteomics and metabolomics, into pdECM-based models, future studies could elucidate the full spectrum of ECM-cancer cell interactions across diverse cancer types. Extending this approach to other malignancies, such as breast or lung cancer, could uncover shared and unique mechanisms of ECM-driven metastasis, providing a more comprehensive understanding of cancer biology.

## Supplementary information

Supplementary Video 1

Supplementary Video 2

Supplementary Video 3

Supplementary Video 4

Supplementary Figures

## Data Availability

All data supporting the findings of this study are available from the corresponding author upon request.
