# Supplementary material for: Single-cell multiomic modelling of early metastatic events promoted by the extracellular matrix
Source: Br J Cancer. 2025 Sep 15;133(11):1708–19. doi: 10.1038/s41416-025-03181-4 (PMC12644670; doi:10.1038/s41416-025-03181-4)
Supplement: Supplementary file 5 — Supplementary Figures [file 41416_2025_3181_MOESM5_ESM.pdf]

## Supplementary Figure 1

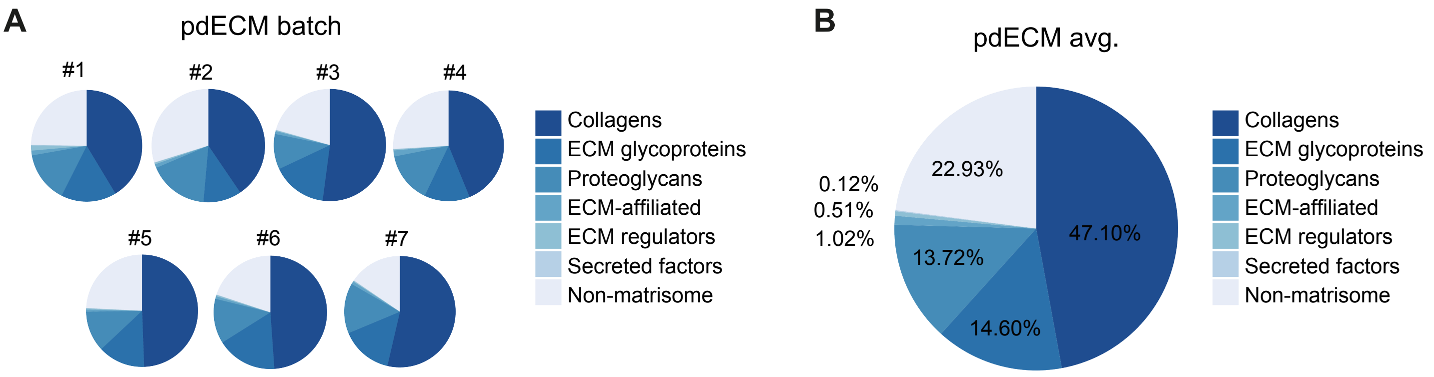

**Supplementary Figure 2**

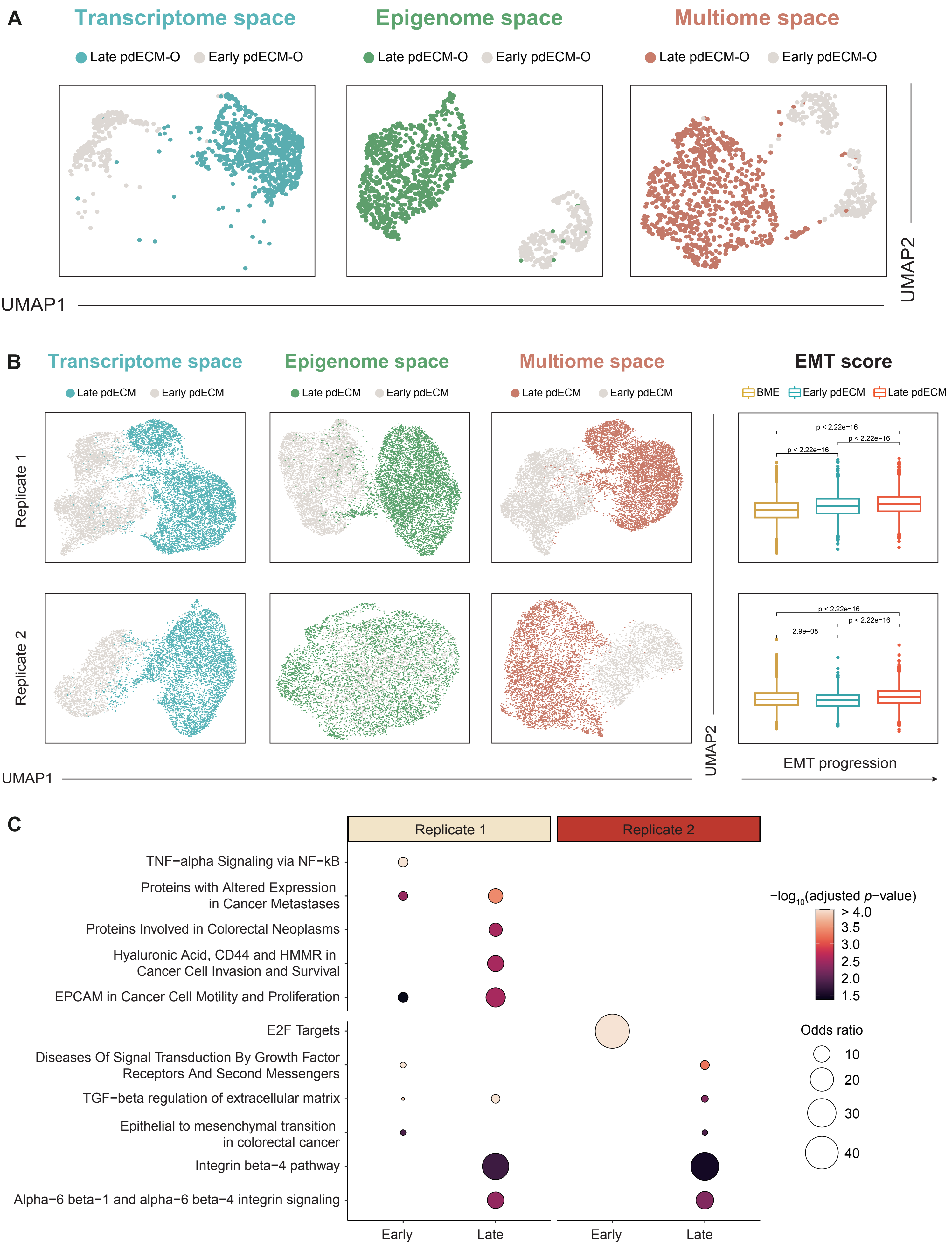

## Supplementary Figure 3

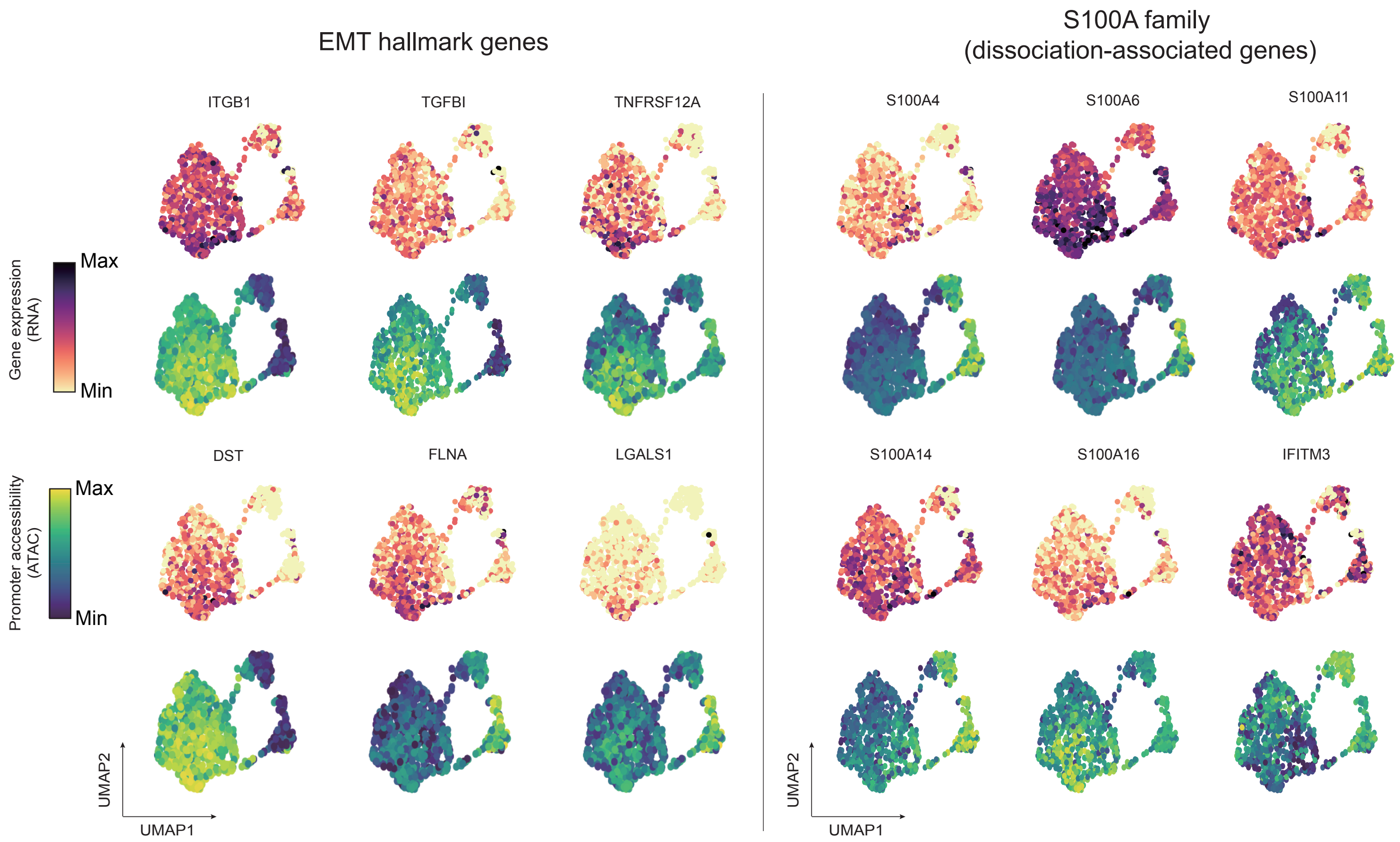

# Supplementary Figure 4

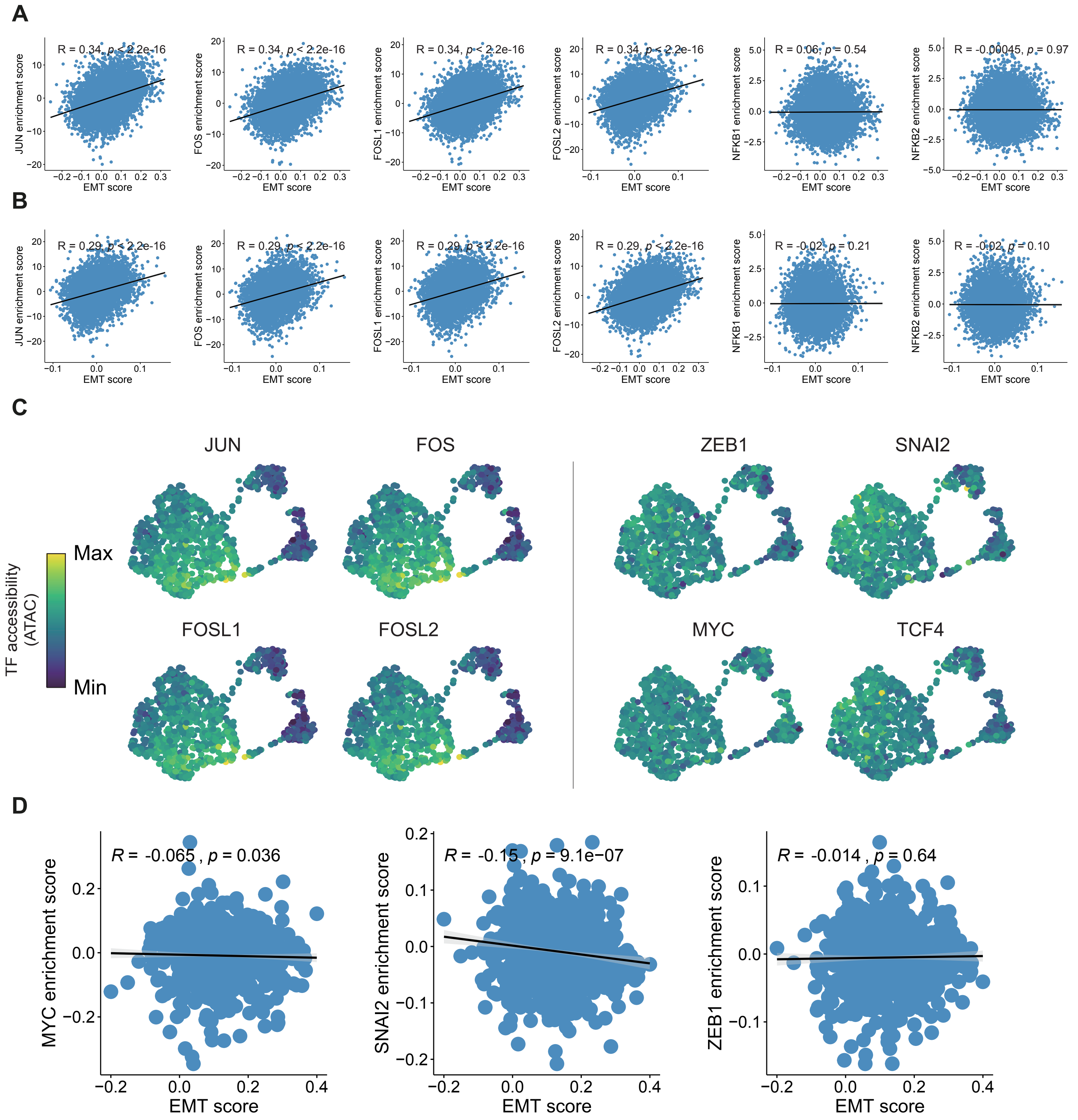

# Supplementary Figure 5

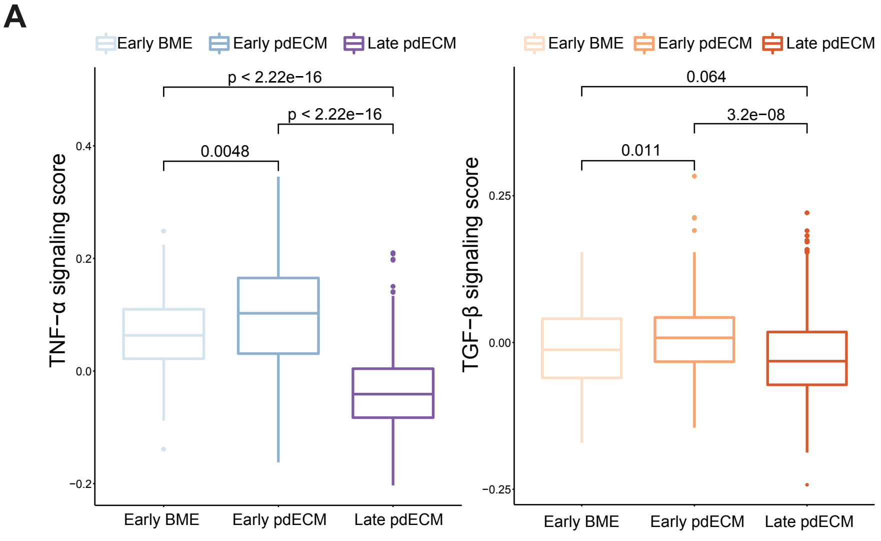


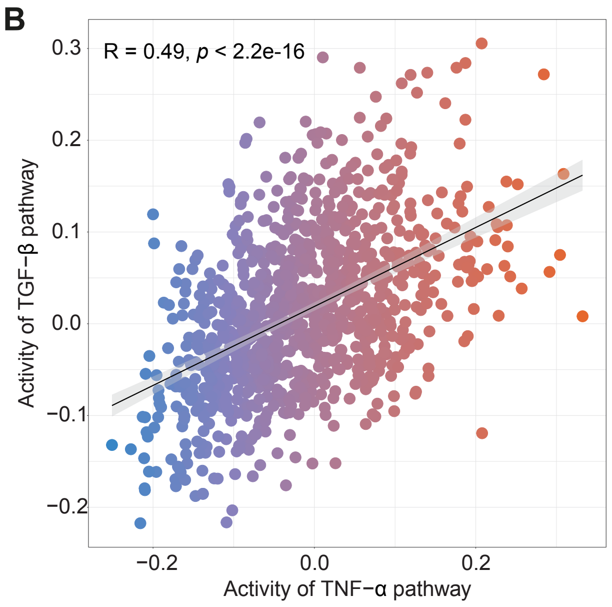

## Supplementary Figure 6

**A**

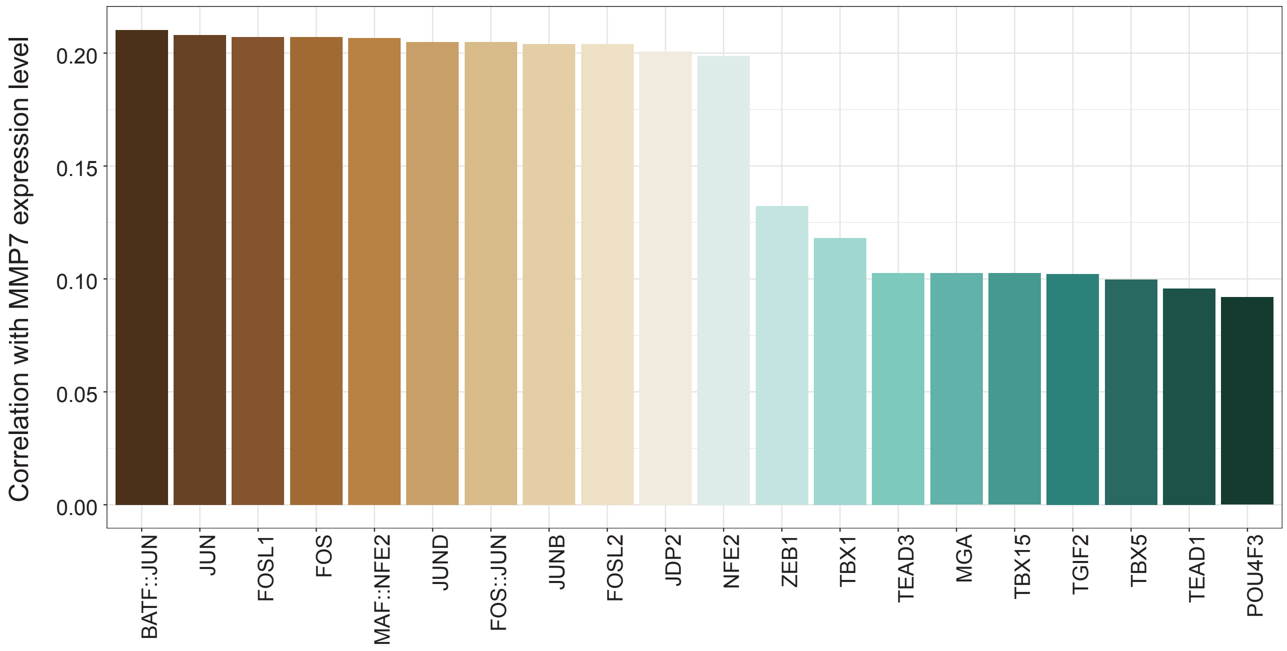

## Supplementary Figure 7

**A**

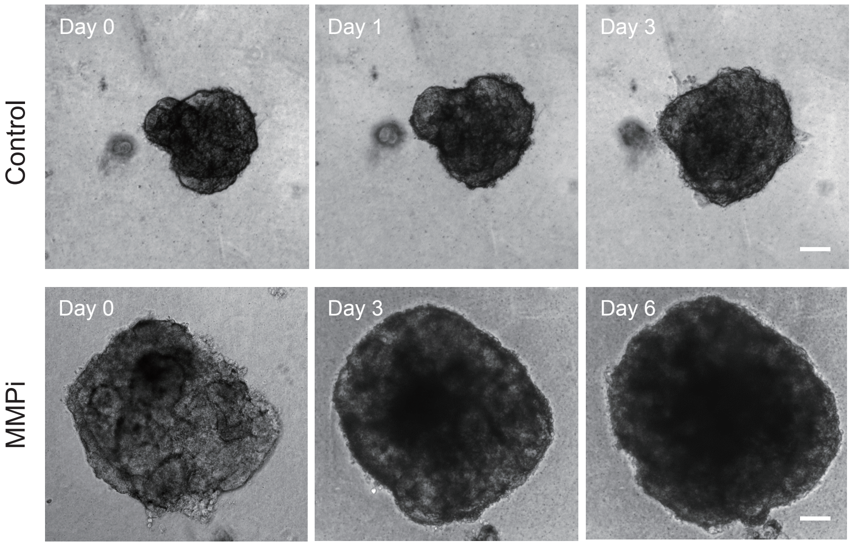

## Supplementary Figure Legends

**Figure S1. | Proteomic analysis of the pdECM** A) Profile of seven normal pdECM samples showing similar compositions of matrisome proteins. B) Average composition of matrisome proteins in the pdECM samples.

**Figure S2. | Single-cell multiomic data of independent biological replicates** A) Projection of the pdECM-O cells in the main experiment onto the transcriptome, epigenome, and multiome UMAP space. B) (left) Projection of the pdECM-O cells of replicate 1 (upper) and replicate 2 (lower) onto the transcriptome, epigenome, and multiome UMAP space. The pdECM-O cells were divided into two clusters (early and late) based on their EMT score. (Right) EMT scores of the BME, early pdECM, and late pdECM cells of replicate 1 (upper) and replicate 2 (lower). Statistical difference in the EMT state was calculated with t-test. C) Biological annotation of genes upregulated in the early and late pdECM cells of replicate 1 and replicate 2 using Enrichr. The color and size of the dots represent adjusted *p*-value and odds ratio, respectively.

**Figure S3. | Expression of EMT hallmark genes and S100A family genes.** Expression and promoter accessibility of EMT hallmark genes (left) and dissociation-associated S100A family (right).

**Figure S4. | Additional TF motif enrichment analyses** A) Spearman correlation between the EMT score and motif enrichment in replicate 1. Black line and grey shade represent the line that best fit the data distribution and confidence interval, respectively. The *p*-value was adjusted with the Benjamini-Hochberg method. B) Spearman correlation between EMT score and motif enrichment of replicate 2. Black line and grey shade represent the line that best fits the data distribution and confidence interval, respectively. The *p*-value was adjusted with the Benjamini-Hochberg method. C) Motif accessibility of the AP-1 complex (left) and EMT master regulators (right) of each cell. D) Spearman correlation between the EMT score and motif enrichment of well-known EMT master regulators. Black line and grey shade represent the line that best fits the data distribution and confidence interval, respectively. Each dot represents a single cell.

**Figure S5. | Co-regulation of TNF-α and TGF- β signaling pathway** A) Enrichment score of TNF-α and TGF-β signaling pathways in the early BME, early pdECM, and late pdECM cells. Statistical differences between the groups were calculated with the t-test. B) Correlation between TNF-α signaling and TGF-β signaling activities. Black line and grey shade represent the line that best fits the data distribution and confidence interval, respectively. Each dot represents a single cell.

**Figure S6. | TFs associated with *MMP7* expression level.** Spearman correlation between TF motif variability and *MMP7* expression level. Only top 20 TFs are shown.

**Figure S7. | The effect of MMP7 inhibition on invasive phenotype.** Optical image of the organoid culture with batimastat showed the decrease of invasive phenotype of the pdECM-O. Scale bar: 100μm.

## Supplementary Movie Legends

Supplementary Video 1. 24 h live optical imaging of BME-cultured organoids

Supplementary Video 2. 15 h live optical imaging of pdECM-cultured organoids

Supplementary Video 3. 7 days tracking optical images of BME-cultured organoids

Supplementary Video 4. 7 days tracking optical images of pdECM-cultured organoids
